# Poster Session II A325 CISPLATIN INDUCES A CONDITIONED TASTE REACTIVITY IN MICE THAT IS MITIGATED BY ONDANSETRON, ELABORATING ON A BEHAVIORAL MODEL TO STUDY CAUSES AND TREATMENTS OF NAUSEA

**DOI:** 10.1093/jcag/gwaf042.325

**Published:** 2026-02-13

**Authors:** V Muranjan, A Vallero, C Nguyen, G Blonde, K Y Hui

**Affiliations:** Internal Medicine, Johns Hopkins University School of Medicine, Baltimore, MD; Internal Medicine, Johns Hopkins University School of Medicine, Baltimore, MD; Internal Medicine, Johns Hopkins University School of Medicine, Baltimore, MD; Florida State University College of Arts and Sciences, Tallahassee, FL; Internal Medicine, Johns Hopkins University School of Medicine, Baltimore, MD

## Abstract

**Background:**

Nausea is a common symptom, yet understanding of its pathogenesis and optimal treatments remains elusive, due partly to limitations in the animal models of nausea. Because rats and mice are non-vomiting species, most animal studies have used other putative nausea-related behaviors. In rats, one such behavioral assay is conditioned taste reactivity (CTR), in which repeatedly pairing a novel taste with a nauseating stimulus yields characteristic aversion-associated movements of the mouth and forelimbs. Relatively few CTR studies have been conducted in mice, however, and none have investigated the effectiveness of nausea treatments.

**Aims:**

We sought to investigate whether clinical nausea is closely modeled by CTR in mice, as it is in rats.

**Methods:**

We prepared 8-12 month old C57B6 mice (n = 64) for CTR testing by implanting intraoral (IO) cannulas. On consecutive days, mice underwent habituation, 5 conditioning sessions, and final testing. During conditioning, 0.1% saccharin was infused IO, immediately followed by intraperitoneal (IP) injection of saline, cisplatin (0.5-2.5 mg/kg body weight), or lithium chloride, a common experimental nausea precipitant. Half the cisplatin- and lithium-treated mice were pre-treated with ondansetron 0.2 mg/kg IP 30 minutes before conditioning. In the testing session, behaviors during saccharin exposure were video recorded and manually scored. For comparison, we also conducted a separate experiment to investigate the effects of ondansetron and 2 other anti-nausea agents using conditioned flavor avoidance (CFA), another putative nausea-related assay focusing on the quantity of saccharin solution freely consumed by cisplatin-exposed mice (n = 15).

**Results:**

In CTR testing, when compared to saline control, cisplatin conditioning at both doses increased the predominant aversive behavior, forelimb flailing (low-dose P = 0.042, high-dose P = 0.027, U test). Mice pre-treated with ondansetron exhibited intermediate levels of forelimb flailing, indicating partial blockade of the cisplatin effect (Fig 1). In CFA testing, all 3 anti-nausea agents, including ondansetron, were ineffective in reducing the effect of cisplatin conditioning.

**Conclusions:**

We showed, for the first time, that cisplatin elicits nausea-like behavior using the CTR assay in mice, and that pre-treatment with ondansetron mitigates this effect. These findings build confidence in the CTR experimental setup, which may be used to study novel nausea precipitants and treatments, as well as transgenic modifications that may elucidate relevant neural pathways.

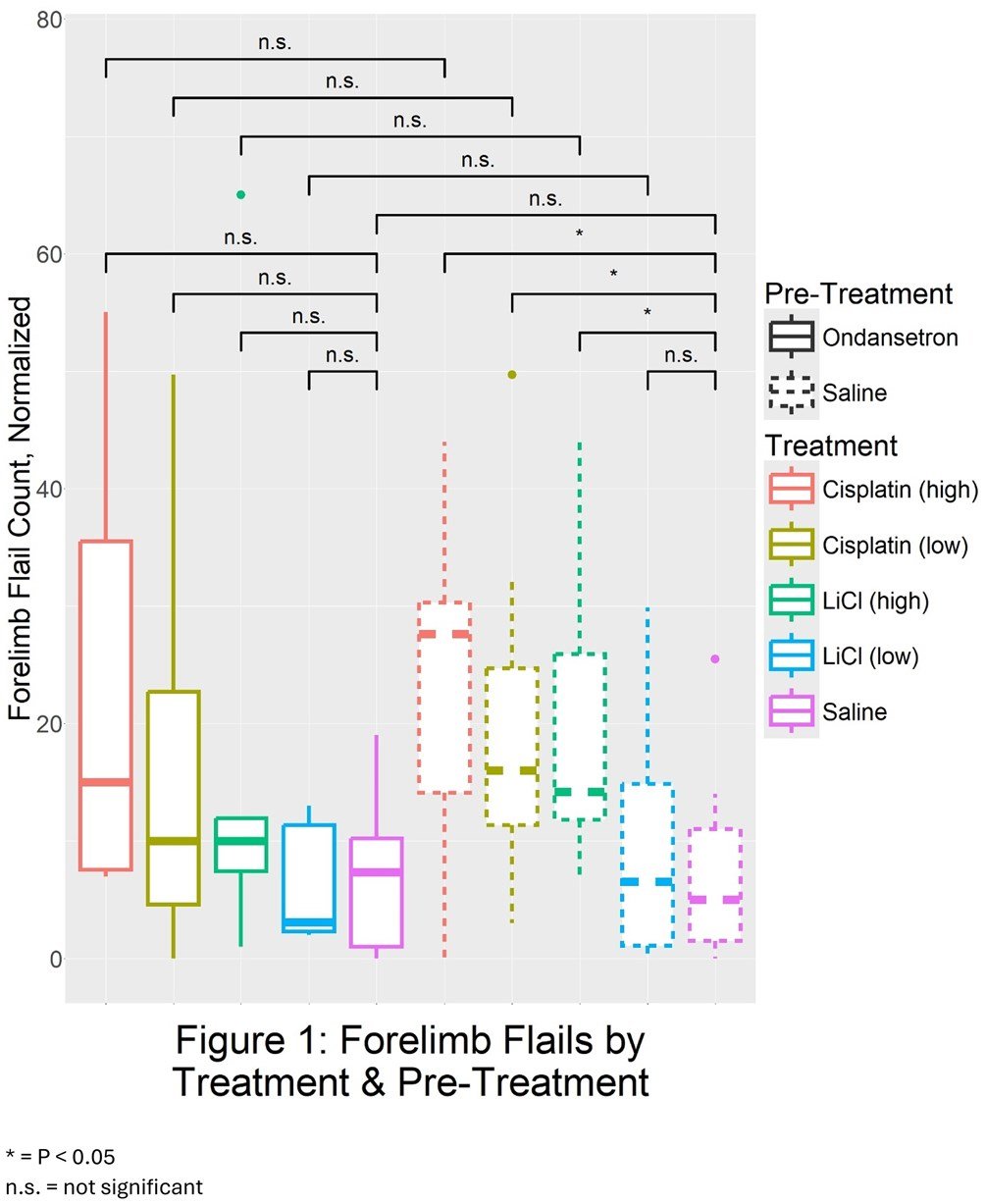

**Funding Agencies:**

None

